# Six co-occurring conifer species in northern Idaho exhibit a continuum of hydraulic strategies during an extreme drought year

**DOI:** 10.1093/aobpla/plz056

**Published:** 2019-09-23

**Authors:** Kathryn V Baker, Xiaonan Tai, Megan L Miller, Daniel M Johnson

**Affiliations:** 1 Department of Forest, Rangeland and Fire Sciences, University of Idaho, Moscow, ID, USA; 2 Department of Environmental Science, Marist College, Poughkeepsie, NY, USA; 3 Department of Geology and Geophysics, University of Utah, Salt Lake City, UT, USA; 4 Warnell School of Forestry and Natural Resources, University of Georgia, Athens, GA, USA

**Keywords:** Hydraulic conductivity, *P*_50_, Pinaceae, safety margin, stomatal conductance, water potential

## Abstract

As growing seasons in the northwestern USA lengthen, on track with climate predictions, the mixed conifer forests that dominate this region will experience extended seasonal drought conditions. The year of 2015, which had the most extreme drought for the area on record, offered a potential analogue of future conditions. During this period, we measured the daily courses of water potential and gas exchange as well as the hydraulic conductivity and vulnerability to embolism of six dominant native conifer species, *Abies grandis*, *Larix occidentalis*, *Pinus ponderosa*, *Pinus monticola*, *Pseudotsuga menziesii* and *Thuja occidentalis*, to determine their responses to 5 months of record-low precipitation. The deep ash-capped soils of the region allowed gas exchange to continue without significant evidence of water stress for almost 2 months after the last rainfall event. Midday water potentials never fell below −2.2 MPa in the evergreen species and −2.7 MPa in the one deciduous species. Branch xylem was resistant to embolism, with *P*_50_ values ranging from −3.3 to −7.0 MPa. Root xylem, however, was more vulnerable, with *P*_50_ values from −1.3 to −4.6 MPa. With predawn water potentials as low as −1.3 MPa, the two *Pinus* species likely experienced declines in root hydraulic conductivity. Stomatal conductance of all six species was significantly responsive to vapour pressure only in the dry months (August–October), with no response evident in the wet months (June–July). While there were similarities among species, they exhibited a continuum of isohydry and safety margins. Despite the severity of this drought, all species were able to continue photosynthesis until mid-October, likely due to the mediating effects of the meter-deep, ash-capped silty-loam soils with large water storage capacity. Areas with these soil types, which are characteristic of much of the northwestern USA, could serve as refugia under drier and warmer future conditions.

## Teaser

As trees photosynthesize, using carbon from the atmosphere to grow and store energy, they also lose water to the air. When soils dry during a drought, continuing to photosynthesize and lose water puts trees at risk of damaging their hydraulic conductivity and overall health. Six conifer species in northern Idaho changed their hydraulic strategies during a record-breaking drought in 2015. All species more tightly regulated their water use in response both to how dry the air was (vapour-pressure deficit) and to how dry the leaves themselves became (leaf water potential) after 2 months without rainfall.

## Introduction

Conifers inhabit some of the most extreme habitats on earth that are capable of supporting tree life forms (Farjon 2008; Brodribb *et al.* 2012). As climate change extends growing seasons, many of these forests will likely experience drought stress earlier, longer and at greater severities. Effects of climate change have been evident in the Pacific Northwest, USA for more than a decade, with rising temperatures and drier summers (Abatzoglou *et al.* 2014). These changes are predicted to continue, with precipitation regimes shifting to greater ratios of rain to snow and temperatures potentially increasing by 4 °C within the 21st century (Mote and Salathé 2010). Increased temperatures will cause the reduced snowpack to melt earlier, creating a positive feedback loop that will further exacerbate extended, xeric growing seasons (Luce and Holden 2009: Klos *et al.* 2014). Characterized by dry summers, the mixed conifer forests of northern Idaho largely depend on soil water storage recharged by snowmelt and springtime precipitation to sustain transpiration throughout the growing season (Baker 1981). Widespread ash-capped soil deposits in the northwestern USA provide high water storage capacity, due to their high porosity and water infiltration, facilitating highly productive forest ecosystems (Kimsey *et al.* 2005). In this region, the deep, ash-capped soils provide mixed conifer forests with adequate soil moisture during the long (3–5 months) dry portion of the growing season (Page-Dumroese *et al.* 2005).

While traditional comparisons of tree species in the region are based on plants’ survival and growth during drought (Daubenmire 1968; Daubenmire and Daubenmire 1968; Minore 1979), another type of framework, degree of isohydry, has been used more recently to describe a continuum of trees’ response to drought (Tardieu and Simonneau 1998; Fu *et al.* 2018; Li *et al.* 2019). Although there have been different definitions, more isohydric species generally control their leaf water potentials (Ψ _leaf_) by decreasing their stomatal conductance (*g*_s_) so that Ψ _leaf_ is maintained above a ‘set point’ (Meinzer *et al.* 2016; Martínez-Vilalta and Garcia-Forner 2017). Alternatively, relatively anisohydric species allow gas exchange and photosynthesis to continue as their Ψ _leaf_ values decline (McDowell *et al.* 2008; Fu and Meinzer 2018). Various measures have been proposed to characterize patterns in species’ water relations, with recent attention given to the regulation of Ψ _leaf_ relating to levels of embolism within the xylem tissue (Meinzer *et al.* 2009; Choat *et al.* 2012; Skelton *et al.* 2015). Stomatal sensitivity to vapour-pressure deficit (VPD), with *g*_s_ declining in response to greater VPDs, has also been related to drought performance (Domec *et al.* 2009; Anderegg *et al.* 2014). However, which parameters are most appropriate for evaluating drought response strategies remains unclear (Hartmann *et al.* 2018).

As climate regimes change and historical species’ distribution areas become potentially unfavourable for continued survival, it is important to address the questions of which tree populations will be prone to mortality and what conditions can facilitate survival (Millar *et al.* 2007; McDowell *et al.* 2016; Mathys *et al.* 2017; Hartmann *et al.* 2018). Local climate plays a large role in driving population success, but other abiotic site characteristics such as favourable soil type and depth can mediate drought conditions (Wei *et al.* 2018). Because few comparisons of hydraulic strategies across regionally dominant conifer species exist (but see Bond and Kavanagh 1999; Piñol and Sala 2000; Pangle *et al.* 2015), our original goal was a general comparative study of several regionally-dominant conifer species: *Abies grandis*, *Larix occidentalis*, *Pinus monticola*, *Pinus ponderosa*, *Pseudotsuga menziesii* and *Thuja plicata*. However, as 2015 proved to be an unusually hot and dry year (Marlier et al. 2017), we took advantage of the unprecedented drought to understand how mixed conifer forests in northern Idaho will fare under future extended seasonal drought conditions. Because this area experiences growing seasons marked by little or no rainfall and high evaporative demand, we expected that these species would exhibit drought-resistant traits such as stomata that are sensitive to VPD, embolism-resistant xylem and positive hydraulic safety margins. In the 1960s, Daubenmire established a wet to dry continuum, characterizing the sites where different tree species are found. For our species, the order on that continuum was: *P. ponderosa* (driest), *P. menziesii*, *L. occidentalis*, *A. grandis*, *P. monticola* and *T. plicata* (Daubenmire 1966, 1968; Daubenmire and Daubenmire 1968; also see Rehfeldt *et al.* 2006 and Franklin and Dyrness 1973).

We hypothesized, based on the positions that these species occupy on Daubenmire’s dry to wet continuum, that they would exhibit a continuum of drought-resistant traits where the species considered most drought tolerant would exhibit (i) the greatest stomatal sensitivity, with *g*_s_ decreasing with higher VPDs and more negative Ψ _leaf_, and (ii) the most embolism resistant xylem and the largest xylem hydraulic safety margins. A plant physiological model, the Terrestrial Regional Ecosystem Exchange Simulator (TREES) model (Mackay *et al.* 2015), was used to evaluate the influence of alternative soil types in mediating species’ response to drought. We hypothesized that (iii) the deep silty-loam soils of the region are important for buffering drought impacts in addition to hydraulic strategies.

## Methods

### Site description

**Figure 1.**
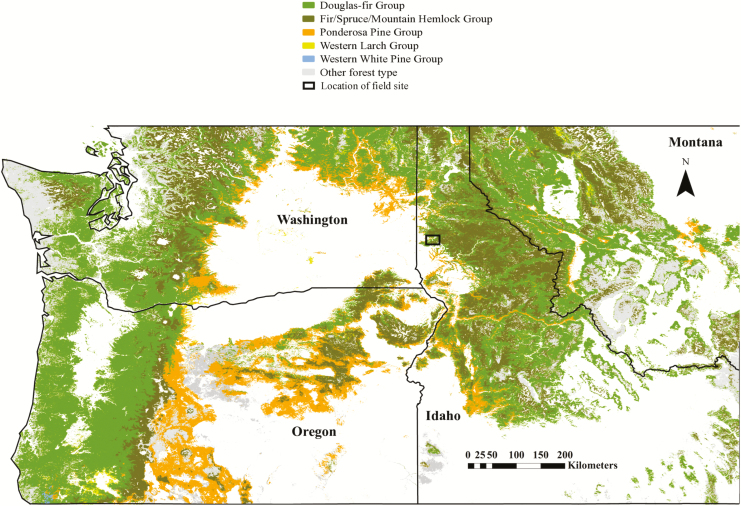
Non-grey highlighted sections represent mixed conifer forests that contain one or more of the study species. The field site is located within the black rectangle in northern Idaho. Forest-type map layer was developed by USDA Forest Service Forest Inventory and Analysis Program and Remote Sensing Applications Center.

This study took place in the Flat Creek section of the University of Idaho Experimental Forest, near Princeton, ID, USA (46°50′/116°43′) (Fig. 1). The area is 0.8 hectares NNE facing with an average slope of 8.5°. The climate is maritime/continental, which is characterized by snowy winters, wet springs and dry summers. The soils are Reggear-Santa complex, an ash cap silty-loam that extends ~1 m in depth (Soil Survey Staff, Natural Resources Conservation Services). For the years 1980–2015, mean annual precipitation was 875 mm; mean annual maximum temperature was 13.5 °C; mean annual minimum temperature was 1.8 °C (Thornton *et al.* 2016).

**Figure 2.**
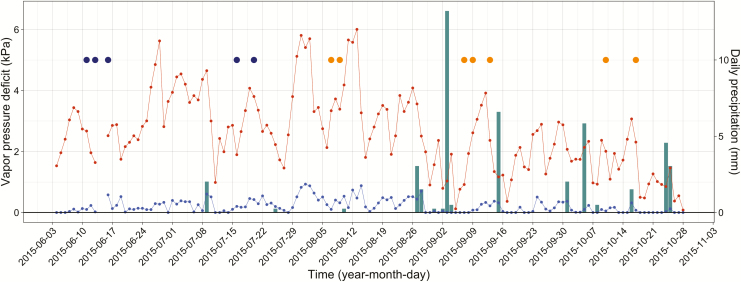
Minimum (blue) and maximum (red) daily VPDs on primary *y*-axis and wet (navy) and dry (orange) sub-season field days with precipitation (bars) on the secondary *y*-axis.

Eight conifer species exist on this single-aged stand, established through a combination of planting and natural regeneration after the site was cleared and burned in 2000. The average spacing between trees was ~3 m. Six of the eight were selected as study species due to their relative abundance: *A. grandis*, *L. occidentalis*, *P. monticola*, *P. ponderosa*, *P. menziesii* and *T. plicata*. As Fig. 1 illustrates, these species comprise a large portion of the forests in the northwestern USA. These six species can co-occur naturally but also span a range of topographical and micro-climatic environments. Ten trees per species were then selected for the study, based on their apparent health, access to sunlight, consistent intraspecific size and range across the site in an effort to keep variation in soil water as similar as possible among species. The trees were between 1.5 and 4 m in height, with diameter at breast height of 5–10 cm and leaves that were easily accessible from the ground. In several cases, individual study trees showed signs of pathogens (i.e. *Cronartium ribicola*, blister rust, in *P. monticola* and *Rhabdocline* spp. fungus in *P. menziesii*) later in the season; in these cases, alternate healthy trees were chosen as replacements. Measurements were performed 2–3 days per month from June to October 2015; for each day (Fig. 2), 3–4 individuals per species were randomly selected from the original 10 for gas exchange and water potential measurements, and no individuals were measured more than once within a month so that 6–10 trees per species were studied each month.

### Meteorological measurements

**Figure 3.**
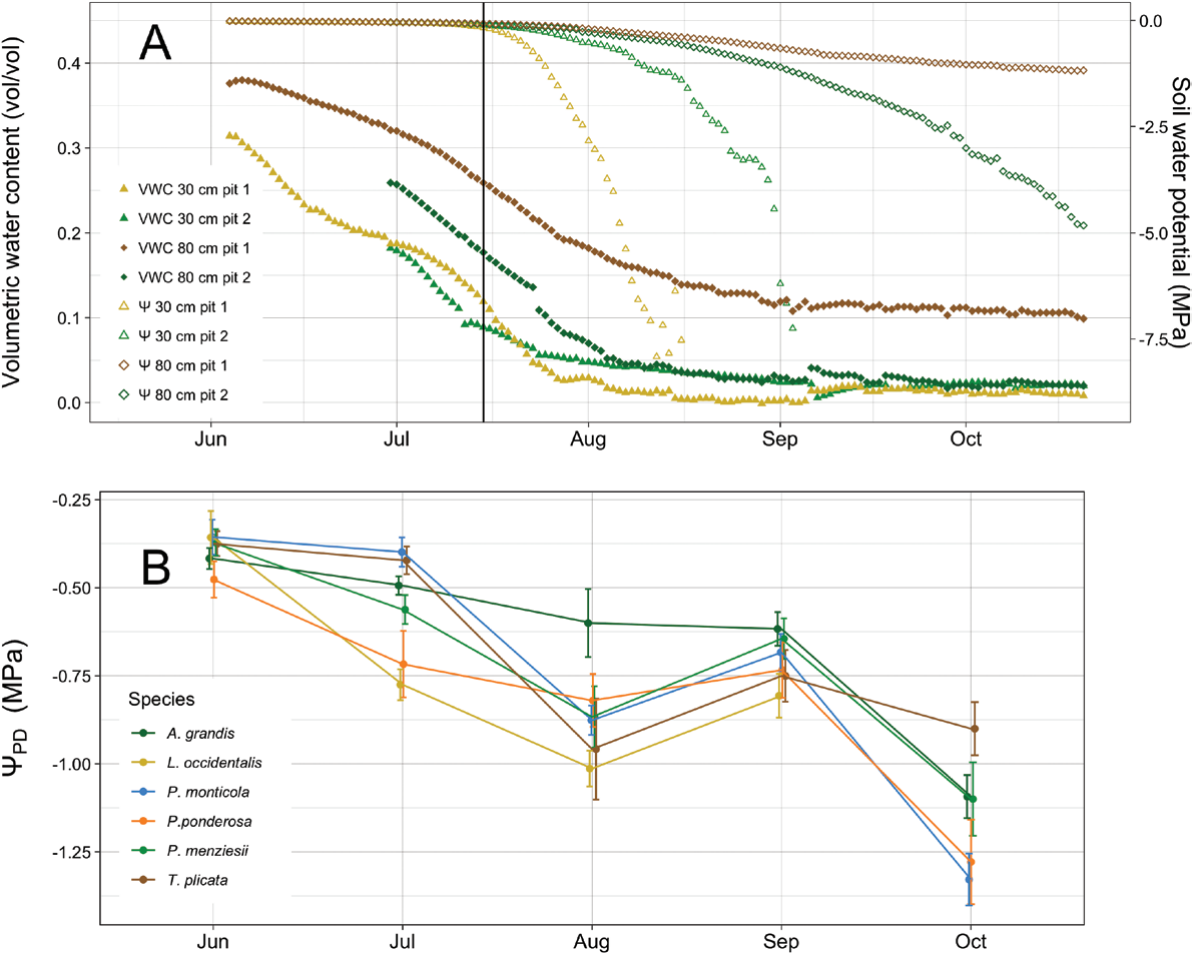
Water potentials of the soil where roots were active declined after >1.5 months without precipitation. (A) Soil water potential and volumetric water content from two positions within field site, with a vertical line positioned 1 day after final July field day to show the partitioning of wet and dry sub-seasons. (B) Predawn water potentials of the six species, averaged within each month. Error bars show standard error.

Volumetric soil water content and soil water potential (VWC and Ψ _S_, GS-1 and MPS-6 sensors, respectively, Decagon Devices, Inc., Pullman, WA) were continuously measured at 30 and 80 cm depths in two soil pits on the site, one in a more densely treed part of the stand and one in a less dense area (pits 2 and 1, respectively, in Fig. 3). Temperature and relative humidity were measured every 5 min and averaged over 30 min (CS-215, Campbell Scientific, Logan, UT), and precipitation was recorded every 30 min using a tipping bucket rain gauge (TE-525, Campbell Scientific). All meteorological sensors were attached to dataloggers (CR-1000T, Campbell Scientific).

### Gas exchange

Gas exchange was measured with a LI-6400 (LI-COR Biosciences, Lincoln, NE) on 3–4 individuals per species per day (6–10 individuals per species per month) every 2 h, beginning at 0600 or 0800 h, depending on time of sunrise, and was repeated every 2 h until 1600 h. For the five species with needle-like leaves, a set number of needles were removed from the tree (five needles for *A. grandis*, *L. occidentalis* and *P. menziesii*, and one fascicle bundle for *P. monticola* and *P. ponderosa*) and placed in the sample chamber, with at least one leaf touching the leaf thermocouple. Previous studies have shown that conifer gas exchange is not affected by removing leaves from the tree in this timeframe (Dang *et al.* 1997; Woodruff *et al.* 2009). Leaves from *T. plicata* were not removed from the tree prior to measurements. The CO_2_ concentration inside the leaf chamber was set to 400 μmol CO_2_ mol^−1^, and flow rate was set to 500 μmol s^−1^; photosynthetically active radiation was set to 1500 μmol m^2^ s^−1^, and ambient temperatures were maintained. To determine leaf area, leaves of six individuals of each species were brought back to the lab each month, trimmed to the LICOR chamber area and measured on the LI-3100C Area Meter. All leaf areas were expressed on a silhouette area basis. The mean of those six samples per species was calculated and used as leaf area for all measurements that month.

### Water potential measurements

Leaf water potential (Ψ _leaf_) was measured using a pressure chamber (Scholander *et al.* 1965; PMS, Albany, OR) beginning well before sunrise, at either 0400 or 0600 h, and was repeated every 2 h until 1600 h. Samples of the youngest hardened needle or branchlet from fully exposed, south-facing branches were clipped and immediately sealed in a bag. Prior to sealing, the air in the bag was humidified by breathing into the bag. The samples were then measured in the pressure chamber within an hour.

In August and September, six total intact branches of each species were covered with plastic bags and aluminium wrap the evening before diurnal measurements. They were sealed to be light- and air-tight to allow their Ψ _leaf_ to equilibrate with branch water potential (Bucci *et al.* 2003). The next afternoon, samples were removed from inside the bags, and their Ψ _leaf_ were recorded for branch Ψ _min_.

### Vulnerability curves

Six root and branch samples were taken of each study species, from trees that were not used for other measurements. Roots were excised from the shallowest available portion of the root system, which was between 20 and 40 cm deep. The root samples were 18–20 cm long when excised with 0.45–1.76 cm diameters after bark was removed. Branches were clipped from the trees, at least 5 cm proximal from the segment to be measured. Their diameters after bark removal were 0.54–1.78 cm. Root and branch samples were immediately placed into black plastic bags with wet paper towels, stored in a cooler and transported to the lab. Samples were cut to 14 cm lengths under water, stripped of their bark and placed in a 20-mM KCl solution (pH = 2) under a partial vacuum overnight. Maximum hydraulic conductivity (*K*_smax_) was determined after overnight vacuuming.

The flow rates of the samples were measured using a plastic tubing manifold with a hydrostatic pressure head of 2.4–6.2 kPa (Sperry *et al.* 1988). After recording the maximum flow rates, negative water potentials were induced in the samples using a centrifuge with submerged sample tips, as described in Alder *et al.* (1997). Care was taken to maintain a balanced centrifuge, adding water into cups, which were 7 mm deep, symmetrically with a pipette to avoid a pressure gradient that would induce pit aspiration (Beikircher *et al.* 2010; Bouche *et al.* 2015). It should be noted that there have been recent debates about the artefacts when measuring vulnerability to embolism (Cochard *et al.* 2010; Sperry *et al.* 2012); however, these artefacts have been predominantly found in long-vesseled angiosperms and not in conifers (Li *et al.* 2008; Cochard *et al.* 2013; Choat *et al.* 2016; Torres-Ruiz *et al.* 2017). Specific hydraulic conductivity (*K*_s_) was calculated by multiplying the flow by the length and dividing by the cross-sectional area of the sample. This process was repeated with progressively more negative water potentials until the flow was <10 % of its maximum value. Measurements were completed within a week of sampling. Branch and root *K*_s_ curves were fit using 3-parameter sigmoidal curves, except *P. monticola* branch *K*_s_, which was fit with a 4-parameter sigmoidal curve because of its greater adjusted *R*^2^ value. *P*_50_ values were subsequently determined from the curves as the estimated water potential at 0.5 *K*_smax_.

### Data analysis

Gas exchange and water potential data were separated into two ‘sub-seasons’, i.e. wet, June–July, and dry, August–October. The sub-seasons were partitioned not based on precipitation but rather on shallow soil Ψ _S_ (Fig. 2). Soil water potentials, measured at 30 cm in two soil pits, remained less negative as volumetric water content declined until late July.

The reported numbers for *g*_s_ and Ψ _leaf_ are the mean hourly values within that month or sub-season. To determine whether species’ stomatal responses to VPD or Ψ _leaf_ were significantly different between sub-seasons, we used linear ANOVA models with sub-season as an interaction term and *post hoc* Tukey’s HSD analyses in R (R Development Core Team 2018) with sub-season as an interaction term (α = 0.05). Vapour-pressure deficit was natural log-transformed to compare linear regression parameters (Oren *et al.* 1999). Regression models, curve fits and confidence intervals were calculated using either R or Sigma Plot 12.5 for Windows (1999, Jandel Scientific Software, San Rafael, CA). Simple and multiple linear regressions were performed in R (R Development Core Team 2018) to address the variables potentially affect *g*_s_, Ψ _leaf_, Ψ _PD_ and VPD. Stomatal conductance and VPD were transformed using a natural log to satisfy normality assumptions.

### TREES model

To compare the influence of different soil types on the study species’ ability to continue transpiring through extended drought, we incorporated our observed physiological traits into the TREES model (Mackay *et al.* 2015). TREES explicitly solves soil-plant hydraulic status following the approach described in Sperry *et al.* (1998), based on half-hourly meteorological forcing data that include air temperature, wind speed, radiation, VPD and soil temperature. It integrates the hydraulic properties of both soil and plant to predict actual transpiration (*E*_c_) and transpiration potential (*E*_crit_).

Terrestrial Regional Ecosystem Exchange Simulator was parameterized for each species based on measured vulnerability to embolism, gas exchange, and predawn and midday leaf water potential observations (Ψ _PD_ and Ψ _MD_). Leaf area index was assumed to be 2 m^2^ m^−2^. Rooting zone was assumed to be 1 m deep and discretized into three layers. Site-specific soil texture data were used to parameterize the soil hydraulic properties (geometric mean particle diameter and geometric standard deviation of particle size), following methods in Campbell (1985). Modelled Ψ _PD_ and Ψ _MD_ values were compared with observations to assess the ability of TREES in capturing the seasonal dynamics of six conifer species **[see****Supporting Information—Fig. S1****]**.

We imposed TREES to different soil types while keeping all other conditions the same and calculated relative hydraulic safety envelopes following the approach described in Johnson *et al.* (2018). For every species and soil type, we calculated the difference between *E*_crit_ and *E*_c_, which is the hydraulic safety envelope. The relative safety envelopes were then determined by normalizing the seasonal mean values by the seasonal maximum. This metric has been used to represent plant vascular health status (Tai *et al.* 2017; Johnson *et al.* 2018).

## Results

### Climate

The temperature, humidity and precipitation of 2015 were compared to the previous 14 years based on data from a nearby SNOTEL site (snow telemetry, Natural Resource Conservation Service; Site Number 989, 1433 m a.s.l.) and to the previous 100 years using National Oceanic and Atmospheric Administration’s (NOAA) Global Historical Climatology Network data for Potlatch, ID (46°54′/116°51′), located 16 km from the field site. Compared to the previous 14 years, the 2015 date of peak snow water equivalent was 43 days earlier than the mean day of 25 March. The ablation date, when the snow is completely melted, was 31 days earlier than the mean of 10 May. Vapour-pressure deficits at the field site ranged from 0.0 to 6.3 kPa. Comparing the dates of our study period (5 June to 17 October) with a 106-year record, 2015 was the third driest year (49.5 mm of rain) with the two drier years being significantly cooler in both mean minimum and maximum temperatures (α = 0.01; **see****Supporting Information—Fig. S2**).

Our field site received even less precipitation than the weather station, with only 27 mm of rain during the study period, and soil water potential measurements from soil sensors showed clear depth-based differences in temporal soil desiccation during the growing season. Water potentials at a depth of 30 cm began to decline at a much greater rate in late July, reaching ca. −8.0 MPa (which is the lower operational limit of these sensors; Decagon, pers. comm.) by mid-August to early September. The water potentials at 80 cm, however, maintained relatively non-negative values during the study period (Fig. 3).

### Gas exchange

**Figure 4.**
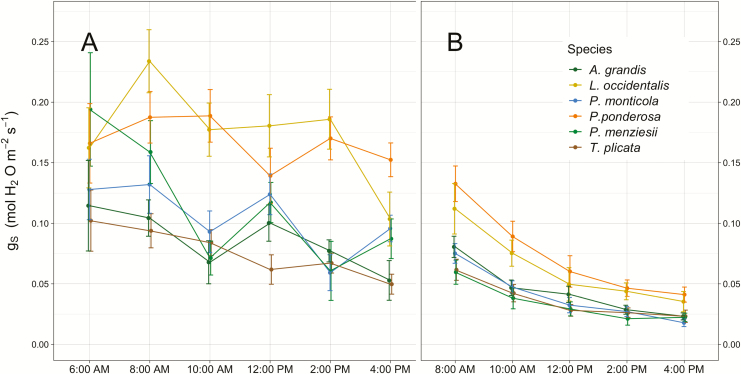
Stomatal conductance, *g*_s_, of each species throughout the day in the (A) wet vs. (B) dry months. Error bars are standard error.

**Figure 5.**
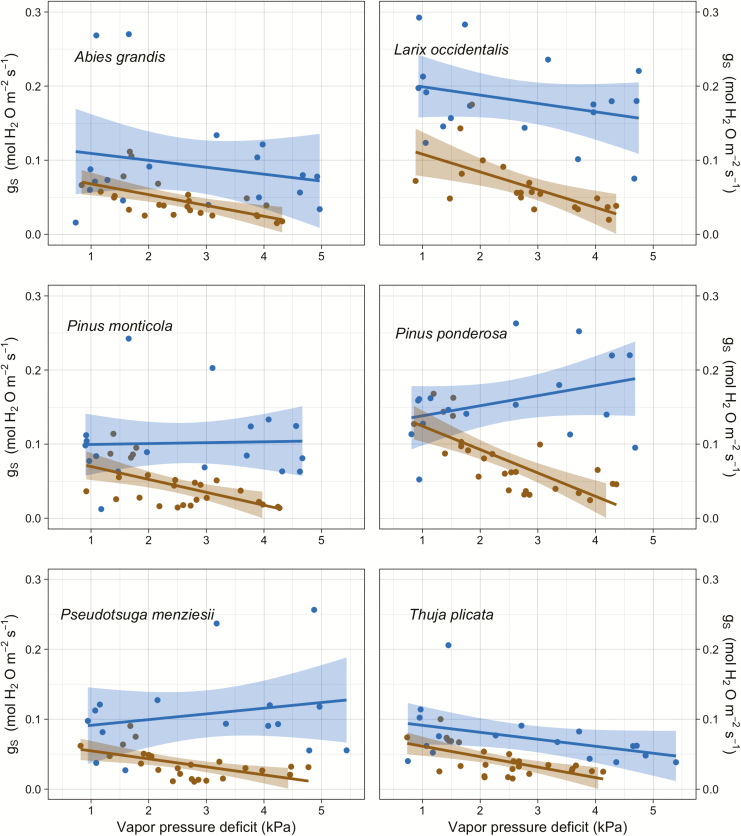
Blue lines and points represent wet sub-season data, and brown lines and points are dry sub-season data. *Larix occidentalis*, *P. monticola* and *P. ponderosa* have significantly different slopes between the dry and wet seasons. *Abies grandis*, *P. menziesii* and *T. plicata* have slopes that are not significantly different from each other.

Stomatal conductance was higher in the wet sub-season compared to the dry sub-season; this comparison was significant for *L. occidentalis*, *P. monticola*, *P. ponderosa* and *P. menziesii* (*P* < 0.05) and was not significant for *A. grandis* and *T. plicata* (*P* = 0.065 and 0.051, respectively). There was greater variation in *g*_s_ as a function of VPD during the wet sub-season, while the dry sub-season *g*_s_ became more consistent throughout the day with less variation (Figs 4 and 5, data points included in **Supporting Information—Fig. S3**). Comparing *g*_s_ to VPD, *L. occidentalis*, *P. monticola* and *P. ponderosa* had significantly different slopes between the two sub-seasons, whereas *A. grandis*, *P. menziesii* and *T. plicata* did not. There was a trend across species of stomata being more sensitive to VPD in the dry sub-season as indicated by the more negative slopes (Fig. 5). In fact, none of the wet sub-season linear regressions of the log of *g*_s_ and the log of VPD (transformed to satisfy normality assumptions) were significant, while all of the same comparison for the dry sub-season were significant (Table 1). Analysing *g*_s_ as a function of Ψ _leaf_, with sub-season as an interaction term, illustrated that only *L. occidentalis* and *P. monticola* had significantly different slopes between sub-seasons, while the dry season again showed generally greater stomatal sensitivity **[see****Supporting Information—Fig. S4****]**.

**Table 1.** Linear regressions relating stomatal conductance to VPD and leaf water potential for the six species in the two sub-seasons. Coefficient *b* was included when *P*-value was <0.05. Vapour-pressure deficit and *g*_s_ were transformed to satisfy normality.

		ln(*g*_s_) = *a* + *b* * ln(VPD)		ln(*g*_s_) = *a* + *b* * |Ψ _leaf_|	
		*b*	Adjusted *R*^2^	*b*	Adjusted *R*^2^
Wet sub-season	*A. grandis*		−0.05		0.17
	*L. occidentalis*		0.04		0.02
	*P. monticola*		−0.04		−0.06
	*P. ponderosa*		0.02		−0.02
	*P. menziesii*		0.00		0.01
	*T. plicata*		0.12		0.15
Dry sub-season	*A. grandis*	−1.09	0.64	−0.93	0.39
	*L. occidentalis*	−1.28	0.63	−1.05	0.28
	*P. monticola*	−1.10	0.45	−0.95	0.20
	*P. ponderosa*	−1.10	0.70	−0.95	0.23
	*P. menziesii*	−0.93	0.46	−0.95	0.49
	*T. plicata*	−0.64	0.27	−0.99	0.35

There was no significant effect of either Ψ _leaf_ or VPD on *g*_s_ during the wet sub-season. In the dry sub-season, however, the same simple linear regressions for both Ψ _leaf_ and the natural log of VPD were both significant for each species. In all cases, lower *g*_s_ was associated with greater VPD and more negative Ψ _leaf_. The results of the multiple linear regressions were more varied, with the incorporation of Ψ _PD_ improving the models in the wet sub-season. Increasing variables generally improved adjusted *R*^2^ values, but coefficients were not necessarily significant **[see****Supporting Information—Table S1****]**.

### Water potentials

For all species, Ψ _PD_ values were significantly more negative in the dry sub-season than in the wet sub-season (Table 2). Only *L. occidentalis* and *P. ponderosa* had significantly distinguishable Ψ _min_ between the two sub-seasons (α = 0.05). *Larix occidentalis* had a more negative Ψ _min_ in the wet sub-season than in the dry, and *P. ponderosa* had a more negative value in the dry sub-season than in the wet. *Larix occidentalis* were not measured in October due to its deciduousity and an early loss of leaves in mid-September.

**Table 2.** Mean Ψ _PD_ and Ψ _min_ for sub-seasons (in MPa; SE in parentheses). Emphasis indicates significant differences for term between sub-seasons. Branch Ψ _MD_ was only recorded in August and September.

	Sub-season	Ψ _PD_	Ψ _min_	Branch Ψ _MD_
*A. grandis*	Wet	**−0.45 (0.03)**	−1.93 (0.22)	
	Dry	**−0.78 (0.11)**	−1.98 (0.10)	−1.27 (0.21)
*L. occidentalis*	Wet	**−0.52 (0.13)**	**−2.71 (0.13)**	
	Dry	**−0.95 (0.04)**	**−2.12 (0.12)**	−1.84 (0.19)
*P. monticola*	Wet	**−0.37 (0.05)**	−1.98 (0.21)	
	Dry	**−0.96 (0.12)**	−1.82 (0.09)	−1.47 (0.06)
*P. ponderosa*	Wet	**−0.61 (0.09)**	**−1.39 (0.18)**	
	Dry	**−0.98 (0.11)**	**−1.95 (0.08)**	−1.47 (0.25)
*P. menziesii*	Wet	**−0.45 (0.06)**	−2.21 (0.21)	
	Dry	**−0.90 (0.07)**	−2.07 (0.06)	−1.55 (0.09)
*T. plicata*	Wet	**−0.39 (0.03)**	−1.63 (0.05)	
	Dry	**−0.82 (0.08)**	−1.72 (0.04)	−1.35 (0.07)

Previous research in northern Idaho has shown that Ψ _PD_ equilibrate with Ψ _S_ on nights with VPDs <0.12 kPa (Kavanagh et al. 2007). Despite consistently large midday VPDs (Fig. 2), the predawn VPDs were low enough to allow the soil and the leaves to equilibrate for half of the field days. Within each week, there were nights with VPDs either above or below the 0.12 kPa threshold. There was not a significant difference in the Ψ _PD_ measurements between those days (*P*-values ranging from 0.29 in *A. grandis* to 0.97 in *P. ponderosa*). Therefore, we considered Ψ _PD_ to be equal to the average Ψ _S_ where the roots are active for this study. An exception to this occurred in September, when the Ψ _PD_ for three species, *L. occidentalis*, *P. ponderosa* and *P. menziesii*, increased to values comparable to those in July (Fig. 3). The September field days occurred just after a 14-mm rainfall event (Fig. 2), which did not affect the Ψ _S_ measured in soil pits and did not appear to infiltrate past the litter layer above the soil (pers. obs.). The increase in Ψ _PD_ may have been due to foliar water uptake (Limm *et al.* 2009; Berry and Smith 2013), but that phenomenon was not addressed in this study.

### Vulnerability curves, isohydry, safety margins and TREES model

**Figure 6.**
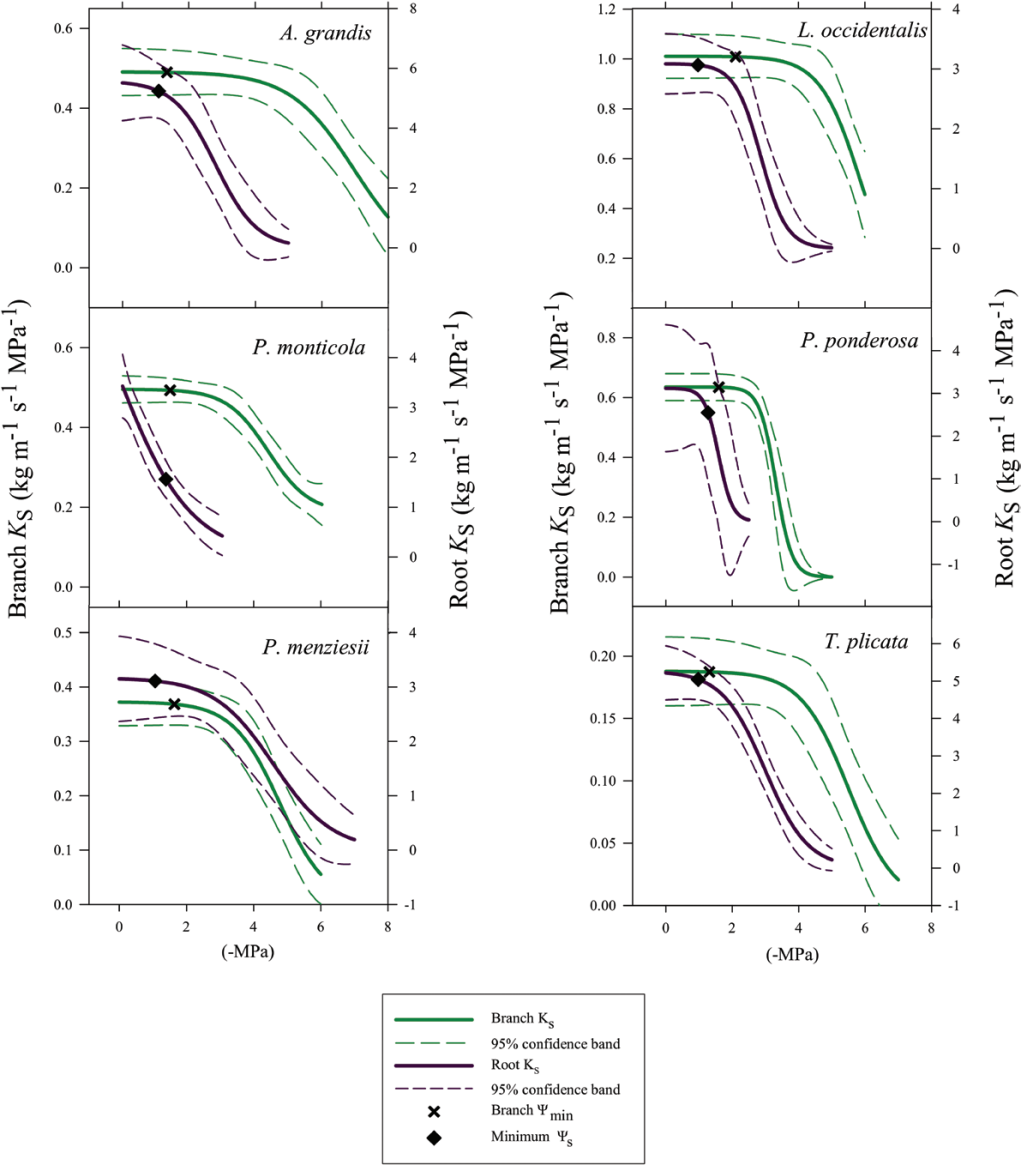
Branch *K*_s_ (solid green lines) and root *K*_s_ (solid purple lines) for the six study species. Thick lines show the regression curve, and dashed lines represent the 95 % confidence interval. Black x’s indicate branch Ψ _min_, and black diamonds show minimum Ψ _S_, which is the most negative predawn water potential by month.

Vulnerability curves and estimated *P*_50_ values for branches and roots (Fig. 6; see Supporting Information—Figs S7 and S8; Table 3) indicated generally large safety margins in those two organs. For all species, branch water potentials remained above the threshold for embolism-induced loss in hydraulic conductivity over the entire study period. For roots, the results were more varied. *Abies grandis*, *L. occidentalis*, *P. menziesii* and *T. plicata* were predicted to lose <6 % of their *K*_s_. The two pines, *P. ponderosa* and *P. monticola*, however, experienced soil water potentials that were predicted to reduce their *K*_s_ by 19 and 56 %, respectively. Using TREES to simulate relative hydraulic safety envelopes in different soil types, silt-loam soil provided the greatest buffer against hydraulic failure for all species. This supported our hypothesis that suitable soil types mediate drought stress.

**Table 3.** Branch and root *P*_50_ values (MPa; confidence intervals in parentheses).

	Branch *P*_50_	Root *P*_50_
*A. grandis*	−7.00 (−7.78, −6.34)	−2.81 (−3.34, −2.25)
*L. occidentalis*	−5.88 (−6.00, −5.53)	−2.87 (−3.24, −2.48)
*P. monticola*	−5.25 (−6.00, −4.73)	−1.30 (−1.49, −0.81)
*P. ponderosa*	−3.32 (−3.59, −3.16)	−1.59 (−2.11, −1.09)
*P. menziesii*	−4.78 (−5.27, −4.34)	−4.59 (−5.44, −3.75)
*T. plicata*	−5.49 (−6.15, −4.84)	−3.00 (−3.28, −2.71)

Using a framework relating *g*_s_ to *P*_50_ and safety margins, all species exhibited isohydric behaviour but to different degrees **[see****Supporting Information—Fig. S5****]**. We determined each species’ *P*_g12_ and *g*_smax_ using a linear model between dry sub-season *g*_s_ and Ψ _leaf_ measurements. The linear models had negative slopes, with *g*_smax_ considered the predicted *g*_s_ at the least negative mean Ψ _leaf_ and *P*_g12_ being the Ψ _leaf_ associated with a *g*_s_ 88 % lower than the *g*_smax_ (Skelton *et al.* 2015). There was a strong positive correlation between degree of isohydry and safety margins across all species (*P* = 0.001; *R*^2^ = 0.986). *Abies grandis* exhibited the largest safety margin along with the highest degree of isohydry while *P. ponderosa* exhibited the lowest safety margin and degree of isohydry. All other species exhibited characteristics between the two extremes. However, using another framework that compared Ψ _min_ to Ψ _PD_, only four species, *A. grandis*, *P. monticola*, *P. menziesii* and *T. plicata*, could be categorized as strictly isohydric. Meanwhile, *P. ponderosa* appeared to be partially isohydric, and *L. occidentalis* falls outside the parameters for categorization (**see****Supporting Information—Fig. S6**; Martínez-Vilalta *et al.* 2014).

## Discussion

### Water potentials and stomatal conductance

Predawn water potential for the six species became more negative as the season progressed, as would be expected during an extended summer drought; however, only *L. occidentalis* and *P. ponderosa* had significantly different Ψ _min_ between the two sub-seasons. Interestingly, *L. occidentalis* exhibited more negative Ψ _min_ during the wet sub-season. Subsequent data from June of 2016 indicated that, for *L. occidentalis*, this may have been due to the morphology of the samples taken (K. V. Baker, unpubl. data, elaboration in **Supporting Information—Text S1**). Leaf water potentials did not decline to values that were measured in the soil at 30 and 80 cm, suggesting that these species likely have access to deeper pools of water.

Our first hypothesis, that all species would have stomatal responses sensitive to VPD, was conditionally supported. In relation to VPD, time of day and Ψ _leaf_, *g*_s_ was more tightly regulated during the dry sub-season than when soil water was more readily available. During the dry sub-season, *g*_s_ decreased to <20 % of *g*_smax_ for all species for most of the day. In the face of extended seasonal drought, this isohydric strategy may lead to decreased carbon stores for these species (e.g. McDowell *et al.* 2008). This trend of having more sensitive stomata in the dry season, which was significantly different from the wet sub-season in *L. occidentalis*, *P. monticola* and *P. ponderosa*, is not entirely explained by the data here. One reason could be the more negative soil water potentials during the dry sub-season; as soil water decreases and becomes more difficult to extract, the plants may adjust their stomatal sensitivity to prevent declines in upstream hydraulic conductivity. This could also be due to changes in leaf turgor during the dry season and/or up-regulation of abscisic acid production which could prime stomata to be more sensitive to dry soils (e.g. Mitchell *et al.* 2016).

The shift in stomatal conductance between wet and dry seasons could also be due to hydraulic capacitance, wherein stored water in the sapwood relieves water stress through the day (e.g. Scholz *et al.* 2011). For this to occur daily, the stored water must be recharged overnight. Mildly negative soil water potentials in the early season may allow the tree to replenish water storage at night, while the dry soil later in the season could prevent recharge (Waring and Running 1978; Meinzer *et al.* 2006, 2009). For the multiple linear regressions in **Supporting Information—Table S1**, adding Ψ _PD_ to the wet sub-season models made the other variables’ coefficients more likely to be significant. This may indicate that capacitive recharge, with greater influence at less negative Ψ _PD_, influenced the trees’ response to other parameters. Continued investigation of capacitive storage in these species is required to determine whether it contributes to dynamic stomatal responses.

### Vulnerability curves, safety margins and TREES model

Many conifer species, including the ones in this study, are on the more isohydric end of the iso/anisohydric continuum (Fu and Meinzer 2018). This can be a successful mechanism for maintaining functionality through a dry period, but it can become harmful during extended drought. Because photosynthetic assimilation ceases after continued stomatal closure, this could eventually lead to carbon reserve depletion (e.g. McDowell *et al.* 2008; Sala *et al.* 2012). Our hypothesis that all six species would fall along a spectrum of isohydry in order of site preference aridity was not supported. There was a strong positive linear correlation between the degree of isohydry and the hydraulic safety margin, which was mainly driven by the species’ very negative *P*_50_ values and the similarity of their *P*_g12_ and Ψ _min_ values across species **[see****Supporting Information—Fig. S5****]**. A similar relationship between stomatal regulation and xylem vulnerability has been seen across 16 species in three California ecosystems where species with more negative minimum leaf water potentials had more resistant xylem (Pivovaroff *et al.* 2018).

Our data are consistent with prior studies describing large hydraulic safety margins in conifers (Choat *et al.* 2012; Johnson *et al.* 2012, 2016). Most of the species in the current study do not have published vulnerability curves for both branches and roots, but those that do, *P. ponderosa* and *P. menziesii*, have similar differences between the organs, with roots being more vulnerable than branches (Domec *et al.* 2006; Johnson *et al.* 2012; Koepke and Kolb 2012; McCulloh *et al.* 2014). As predicted, all species’ xylem had positive safety margins with the exception of the two *Pinus* species’ roots, which were predicted to lose some hydraulic conductivity. All species maintained branch water potentials much less negative than their respective *P*_50_ values (Table 2). This safety margin was greatest in *A. grandis* and least in *P. ponderosa*, contradicting our hypothesis that the species most traditionally accepted as drought tolerant would also have the greatest safety margins. *Pinus ponderosa*, which is well-established as a xeric species (Minore 1979), is the least isohydric of the six species according to Skelton *et al.*’s (2015) metrics for calculating isohydry and degree of safety. Meanwhile, *A. grandis*, a mesic species, was the most isohydric and most conservative in terms of branch and root xylem safety margins, which is consistent with another study in the region (Piñol and Sala 2000). It is worth noting that many other plant physiological parameters including allometry (Lines *et al.* 2012), rooting depth (Padilla and Pugnaire 2007) and hydraulic capacitance (Barnard *et al.* 2011) may be responsible for species drought tolerances and by looking only at xylem vulnerability curves, we may be missing part of the picture.

**Figure 7.**
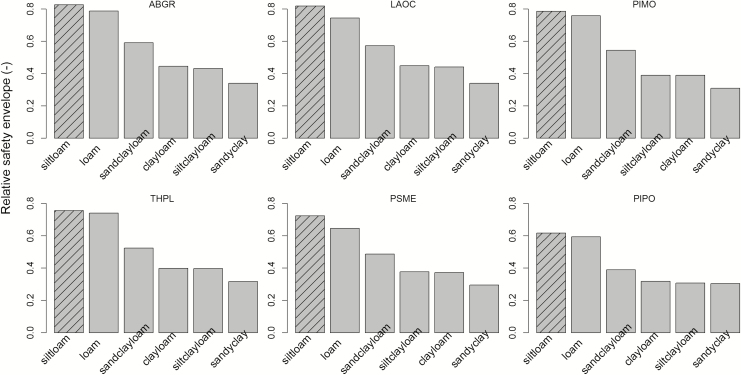
Relative safety envelope of six species throughout the 2015 growing season associated with different soil types. Bars with hatches were the soil type of the current study site.

Based on TREES simulations, each species exhibited higher relative hydraulic safety envelopes in silt-loam compared to alternative soil types (Fig. 7). This indicates that the soils at our site provided a buffer against the severe atmospheric drought and also helps to explain the observed delay in drought stress responses, such as tighter stomatal regulation. This is consistent with earlier suggestions that plant response to drought could be limited by both xylem traits and the rhizosphere (Sperry *et al.* 1998). Our species’ limitations appear to be primarily underground: soil water being ultimately limiting for all species and root embolism starting to affect conductivity in the *Pinus* species.

### Conclusions

The study period of 2015 was the most severe summer drought in the region on record with combined high temperatures and low precipitation. Our findings provide insights into these conifer species’ resilience to extended seasonal droughts that are predicted for this region. Their high stomatal conductance in the early season allowed them to assimilate carbon when soil water was abundant, and their greater regulation during the dry sub-season prevented embolism in their branches and roots (with the exception of *Pinus* roots). If longer growing season droughts occur, these strategies may not be as successful, especially for the species that exhibited some degree of root embolism (*Pinus*). It is important that models predicting plant performance under future climate scenarios take roots into account, as roots can drive loss of whole-plant hydraulic conductance in instances of their greater vulnerability. While the 2015 season was an extreme drought, there had been 122 mm of rainfall that recharged the soil over the 3 weeks directly preceding our study; this rain followed more than a month with negligible precipitation (total of 15 mm), with the last freeze occurring in mid-April (snow telemetry, Natural Resource Conservation Service; Site Number 989, 1433 m a.s.l.). Without the rainfall in May, the 2015 drought would have been 2 months longer. While a drought of that magnitude would certainly cause more water stress, these trees have an advantage over most montane forests: a meter of silty-loam soil that stores water long after precipitation ceases. The ash-capped soils of this region may allow the established forests to persist as their growing seasons become longer, warmer and drier.

## Data

All data analysed in this paper has been made available at www.datadryad.org.

## Supporting Information

The following additional information is available in the online version of this article—

**Figure S1.** Predicted (lines) and observed (dots) Ψ _PD_ and Ψ _MD_ over the course of growing season in year 2015. Red colours represent midday (1200–1400 h), and blue colours represent predawn (0400–0600 h).

**Figure S2.** Weather data from a long-term National Oceanic and Atmospheric Administration (NOAA) meteorological station in Potlatch, ID. The red star represents the year of 2015. Data are from the Julian days of the study period in 2015 for each year.

**Figure S3.** Blue dots and lines represent wet sub-season data, and brown dots and lines are dry sub-season data. Each data point represents the mean values of 3–4 trees on the same day.

**Figure S4.** Blue lines represent wet sub-season data, and orange lines are dry sub-season data. *Larix occidentalis* and *P. monticola* have significantly different slopes between the dry and wet seasons (α = 0.05). *Abies grandis*, *P. ponderosa*, *P. menziesii* and *T. plicata* have slopes that are not significantly different from each other. Data points represent hourly means of 3–4 trees.

**Figure S5.** *Y*-axis is degree of isohydry, defined as *P*_g12_ − *P*_50_; *x*-axis is safety margin, Ψ _min_ − *P*_50_, as described in Skelton *et al.* (2015). *P*_g12_ is the Ψ _leaf_ at which the *g*_s_ is 12 % of *g*_smax_. Ψ _min_ is the most negative Ψ _leaf_ observed. *R*^2^ = 0.986. Dashed line is 1:1.

**Figure S6.** Each data point represents the mean values of 3–4 trees on the same day. *R*^2^ values are listed in legend.

**Figure S7.** Data points represent the *K*_s_ of a single branch (green triangle) or root (purple circle).

**Figure S8.** Data points represent percent loss of conductivity of a single branch (green triangle) or root (purple circle).

**Table S1.** Coefficients of simple and multiple linear regressions shown are significant at *P* < 0.05. The data sets used are listed on the left. Data ‘averaged within days’ consist of means of 3–4 trees’ parameters within species each hour. ‘Not averaged’ data occur only in dry sub-season and relate each individual tree’s vapour-pressure deficit (VPD), leaf water potential (Ψ _leaf_) and/or predawn water potential (Ψ _PD_). Vapour-pressure deficits were in kPa; Ψ _leaf_ and Ψ _PD_ values were in bars; and *g*_s_ were in mol m^−2^ s^−1^.

**Text S1.** Samples of mature shoots.

plz056_suppl_Supplementary_Material

## Sources of Funding

This work was supported by funding from the National Science Foundation (#IOS-1146746). This material is based upon work that is supported by the National Institute of Food and Agriculture, United States Department of Agriculture, McIntire Stennis project under 1004149. K.V.B. was supported by fellowships from the Northwest Climate Science Center and the Stillinger Trust.

## Contributions by the Authors

D.M.J. and K.V.B. designed the project. KVB and MLM performed research, with M.L.M. programming dataloggers for continuous measurements. K.V.B. and D.M.J. analysed data. XT modeled the data. K.V.B., D.M.J., X.T. and M.L.M. wrote the paper.

## Conflict of Interest

None declared.
