# Supplementary material for: Six co-occurring conifer species in northern Idaho exhibit a continuum of hydraulic strategies during an extreme drought year
Source: AoB Plants. 2019 Sep 23;11(5):plz056. doi: 10.1093/aobpla/plz056 (PMC6804486; doi:10.1093/aobpla/plz056)
Supplement: plz056_suppl_Supplementary_Material [file plz056_suppl_supplementary_material.pdf]

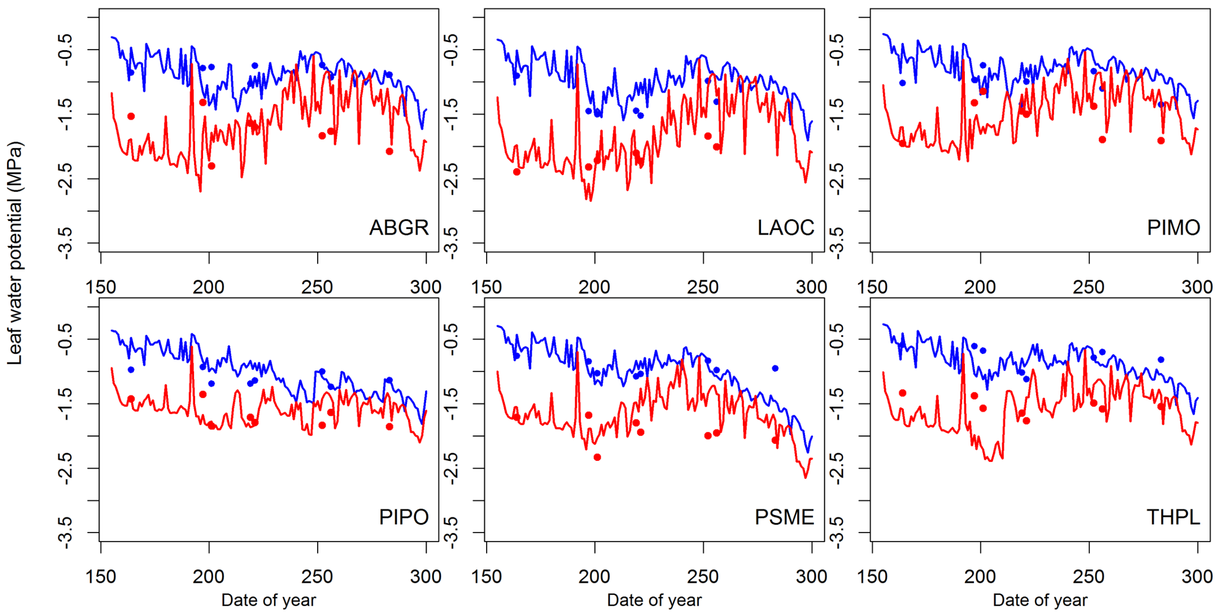

Supplemental Figure 1. Predicted (lines) and observed (dots) Ψ_{PD} and Ψ_{MD} over the course of growing season in year 2015. Red colors represent midday (12:00-14:00), and blue colors represent predawn (4:00-6:00).

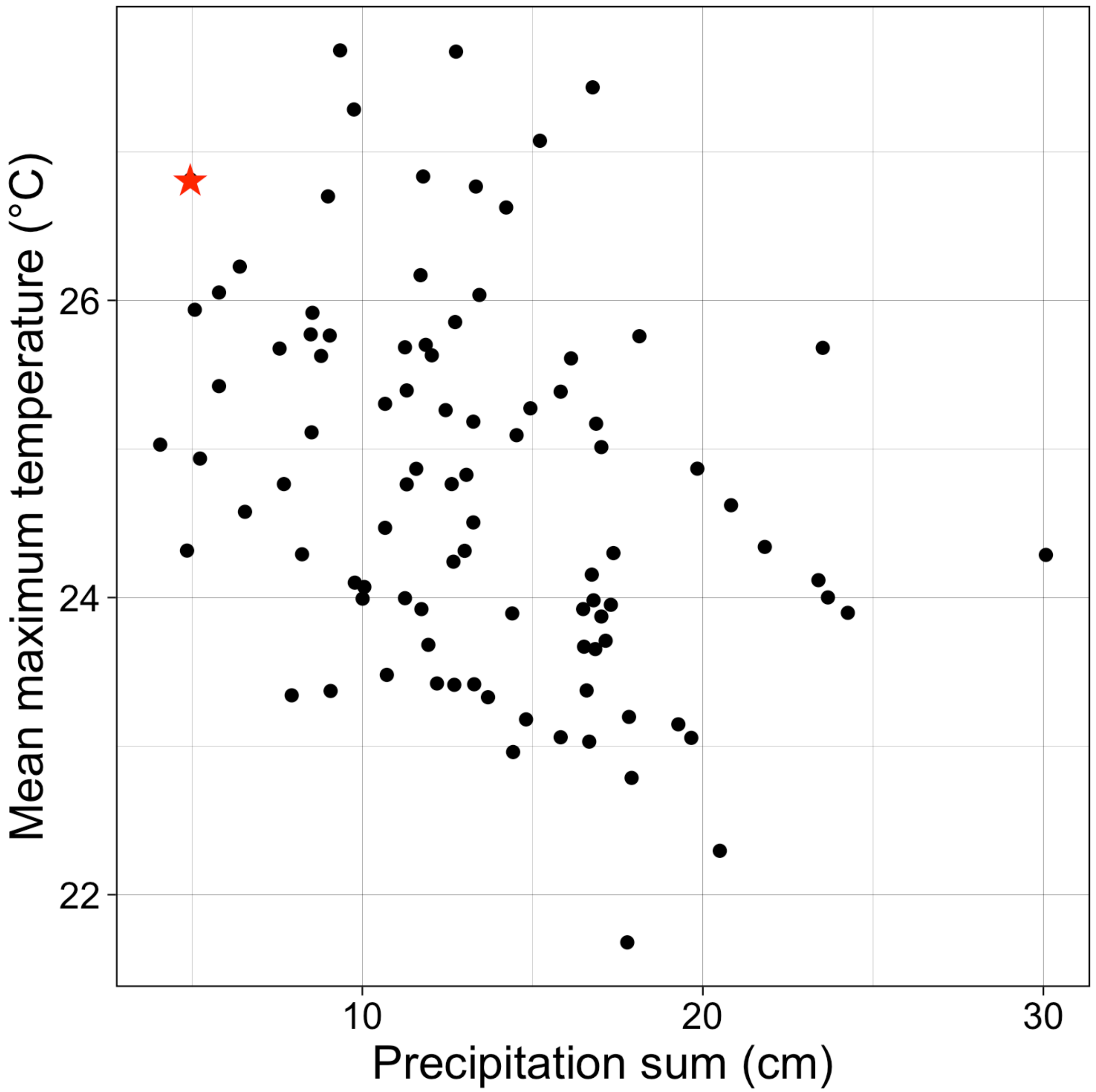

Supplemental Figure 2. Weather data from a long-term NOAA meteorological station in Potlatch, ID. The red star represents the year of 2015. Data is from the Julian days of the study period in 2015 for each year.

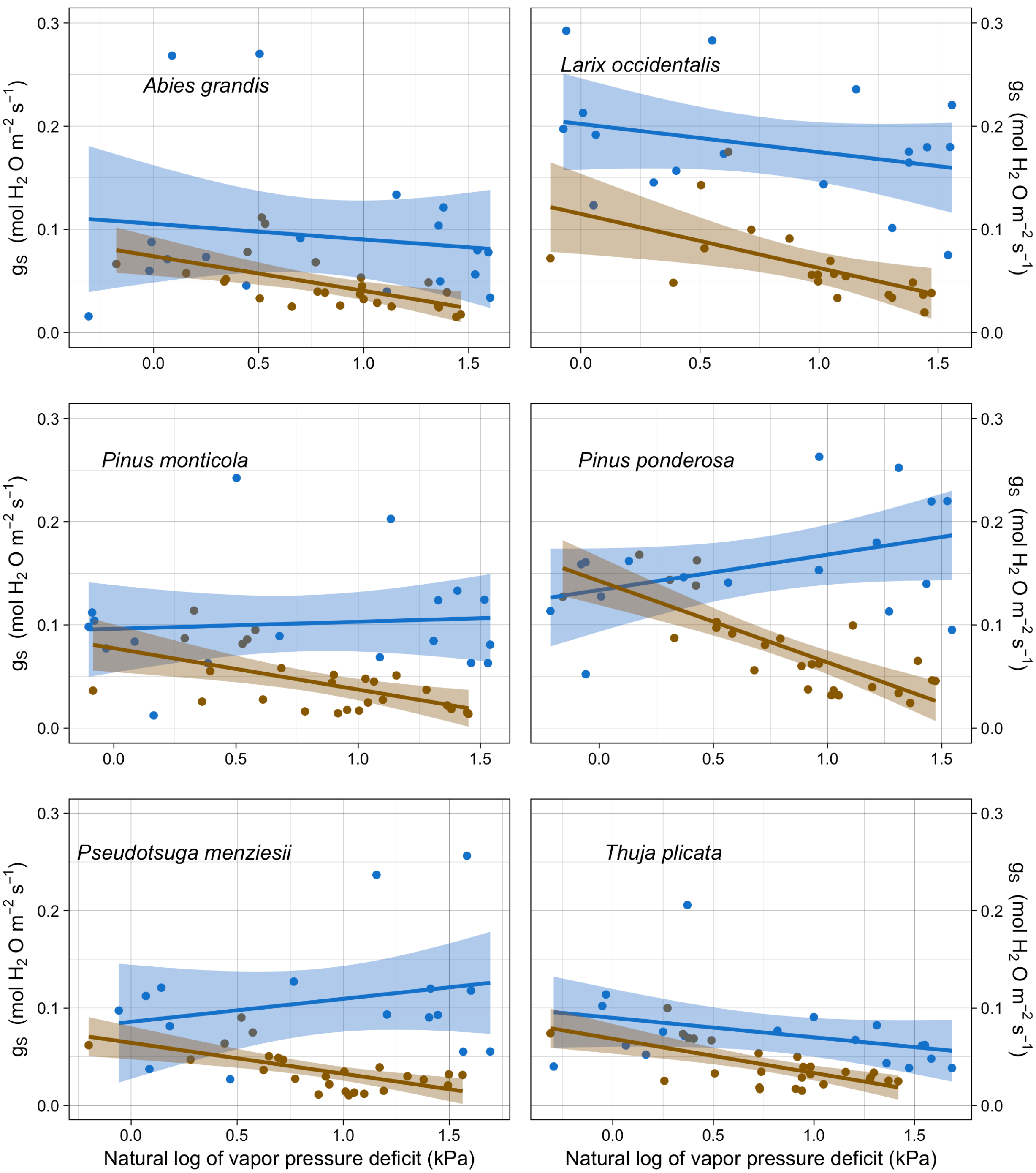

Supplemental Figure 3. Blue dots and lines represent wet sub-season data, and brown dots and lines are dry sub-season data. Each data point represents the mean values of 3-4 trees on the same day.

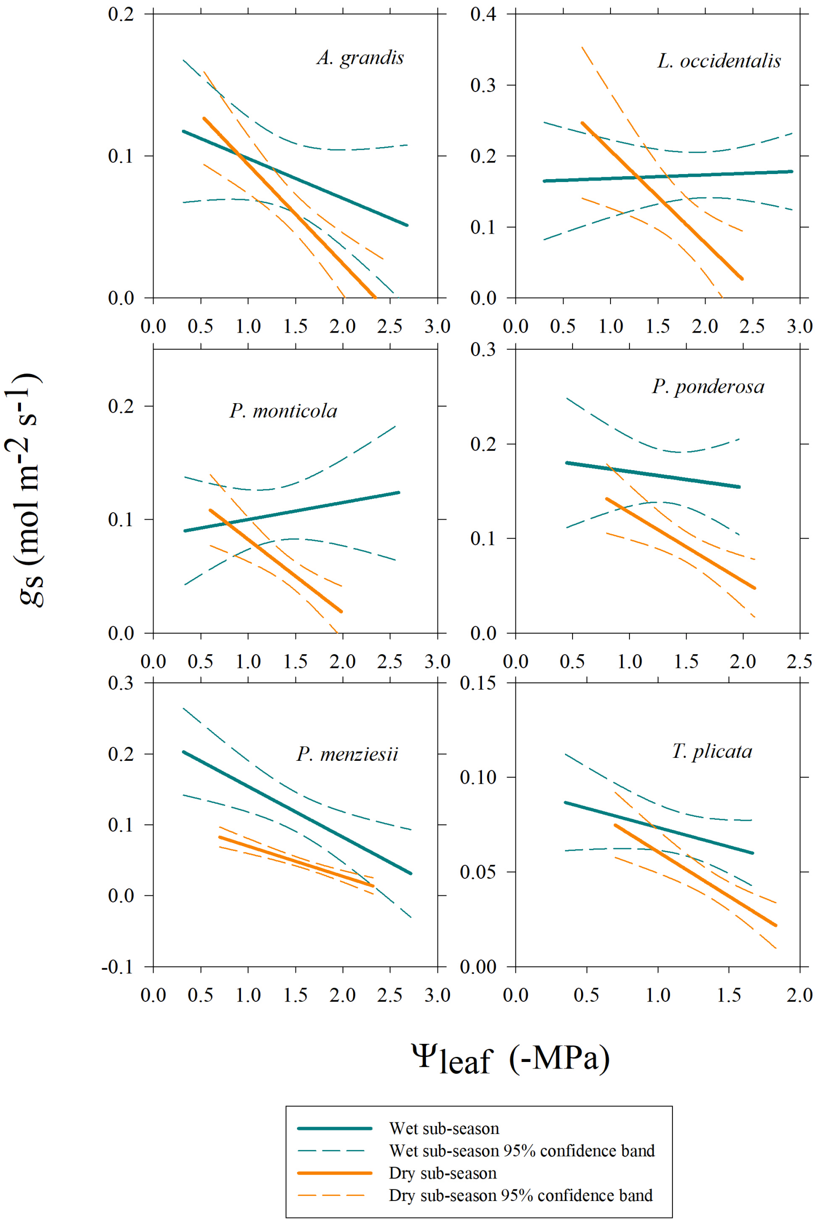

Supplemental Figure 4. Blue lines represent wet sub-season data, and orange lines are dry sub-season data. *L. occidentalis* and *P. monticola* have significantly different slopes between the dry and wet seasons ($\alpha = 0.05$). *A. grandis*, *P. ponderosa*, *P. menziesii*, and *T. plicata* have slopes that are not significantly different from each other. Data points represent hourly means of 3-4 trees.

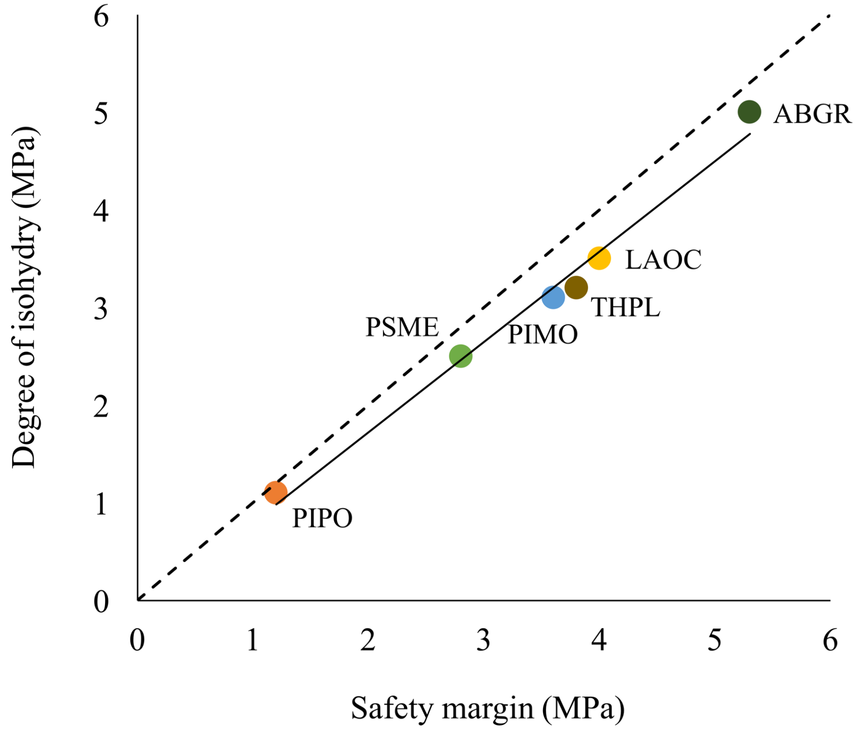

Supplemental Figure 5. Y-axis is degree of isohydry, defined as $P_{g12} – P_{50}$; x-axis is safety margin, $\Psi_{min} – P_{50}$, as described in Skelton *et al*. (2015). P_{g12} is the Ψ_{leaf} at which the g_s is 12% of g_{smax}. Ψ_{min} is the most negative Ψ_{leaf} observed. R^2=0.986. Dashed line is 1:1.

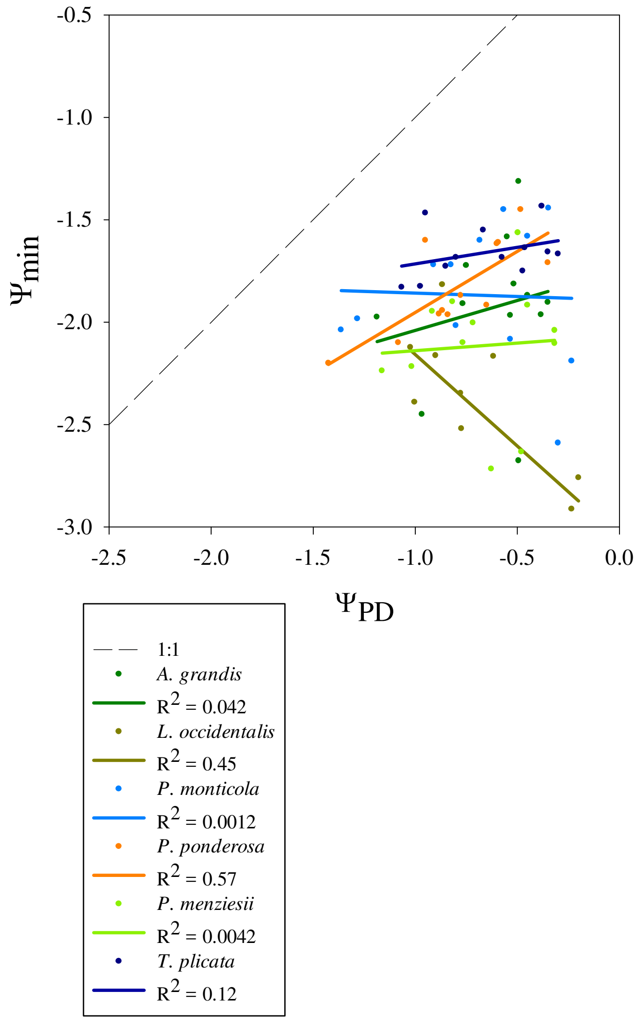

Supplemental Figure 6. Each data point represents the mean values of 3-4 trees on the same day. R^2 values are listed in legend.

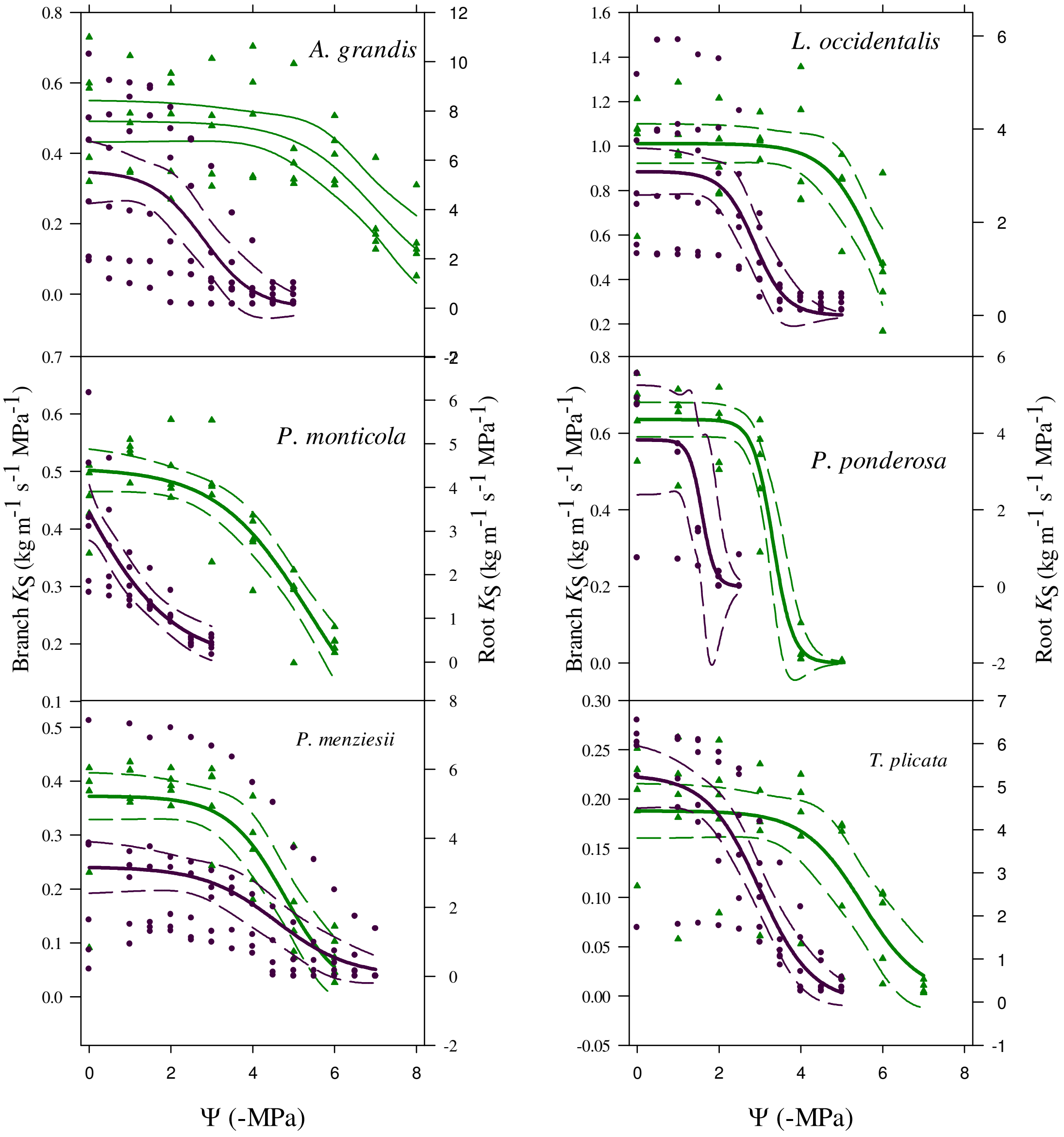

Supplemental Figure 7. Data points represent the K_s of a single branch (green triangle) or root (purple circle).

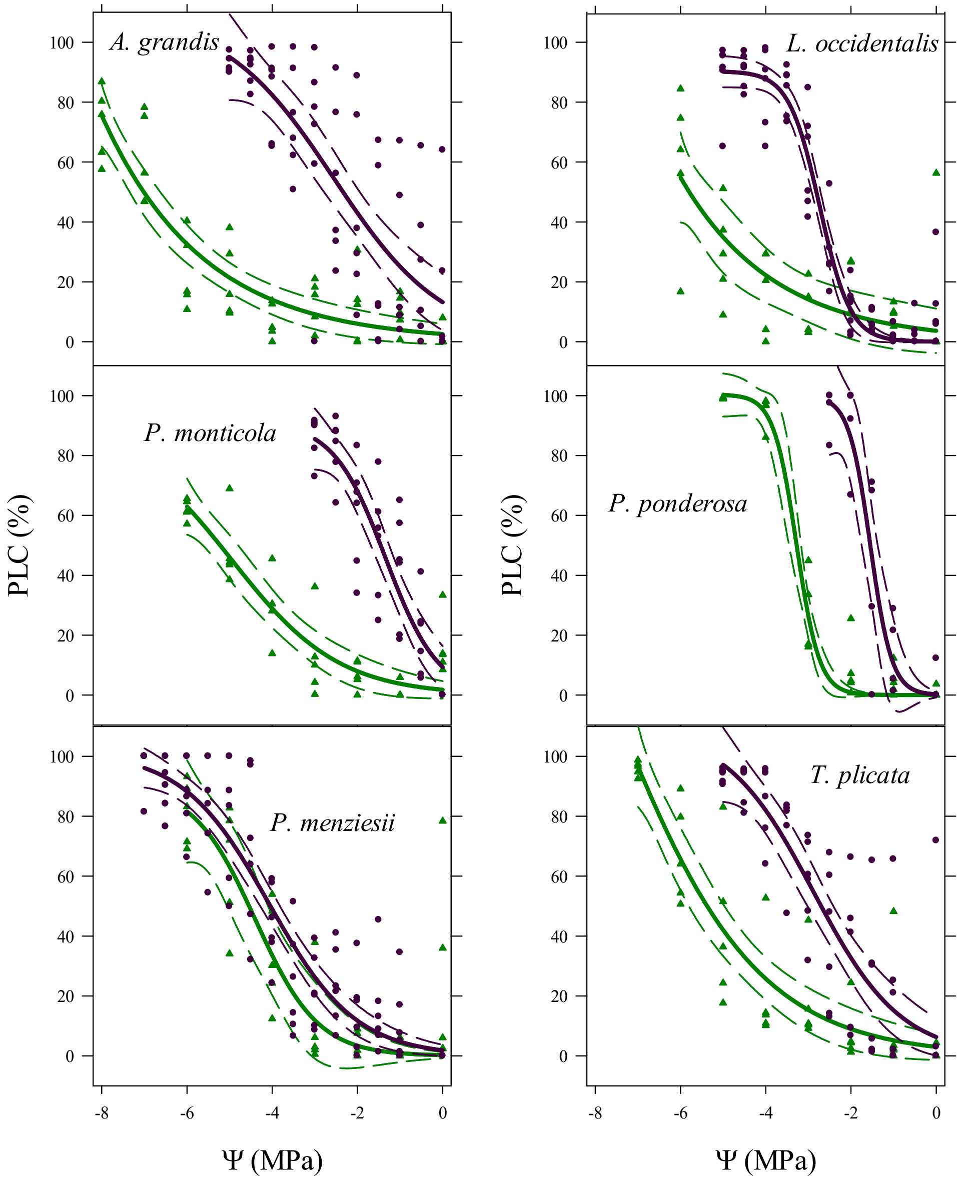

Supplemental Figure 8. Data points represent percent loss of conductivity of a single branch (green triangle) or root (purple circle).

	model	$\ln(gs)=a+b*\ln(VPD)$		$\ln(gs)=a+b*\lvert\Psi_{leaf}\rvert$		$\ln(gs)=a+b*\lvert\Psi_{leaf}\rvert+c*\ln(VPD)+d*(\lvert\Psi_{leaf}\rvert:\ln(VPD)$				$\ln(gs)=a+b*\lvert\Psi_{PD}\rvert+c*\ln(VPD)+d*(\lvert\Psi_{PD}\rvert:\ln(VPD))$				$\ln(gs)=a+b*\lvert\Psi_{PD}\rvert+c*\lvert\Psi_{leaf}\rvert+d*(\lvert\Psi_{PD}\rvert:\lvert\Psi_{leaf}\rvert)$				$\ln(gs)=a+b*\lvert\Psi PD\rvert+c*\lvert\Psi leaf\rvert+d*\ln(VPD)+e*(\lvert\Psi_{PD}\rvert:\lvert\Psi_{leaf}\rvert)+f*(\lvert\Psi_{PD}\rvert:\lvert\ln(VPD))+g*(\lvert\Psi_{leaf}\rvert:\ln(VPD))+h*\lvert\Psi_{PD}\rvert:\lvert\Psi_{leaf}\rvert:\ln(VPD$							
	species	b	adjusted R^2	b	adjusted R^2	b	c	d	adjusted R^2	b	c	d	adjusted R^2	b	c	d	adjusted R^2	b	c	d	e	f	g	h	adjusted R^2
Wet and dry sub-seasons, averaged within days	ABGR	-0.499	0.161	-0.687	0.284	-0.673			0.266				0.472		-1.044		0.390								0.496
	LAOC	-0.688	0.240		0.036				0.220	-2.413	-0.984		0.736				0.454								0.722
	PIMO	-0.417	0.065	-0.609	0.095	-1.100	-1.387		0.156			-1.910	0.512				0.288								0.499
	PIPO	-0.440	0.117	-0.861	0.197				0.194		1.032	-2.067	0.465				0.330					-8.868			0.512
	PSME		-0.012	-0.767	0.175	-1.349			0.204		1.710	-3.026	0.272				0.261								0.689
	THPL	-0.374	0.106	-0.891	0.340	-0.646			0.332				0.282				0.370			3.161		-5.995	-2.282	4.064	0.392
Wet sub-season, averaged within days	ABGR		-0.049		0.169				0.197	-16.897	-4.018		0.563	-10.600			0.452								0.427
	LAOC		0.044		0.020				-0.023				0.448				0.070								0.381
	PIMO		-0.041		-0.060				-0.132	-6.344			0.395				0.192								0.195
	PIPO		0.019		-0.016		1.494		0.334				0.327				0.310								0.239
	PSME		-0.002		0.014				0.216				-0.044				-0.019								0.037
	THPL		0.123		0.149			0.038	0.326				0.197				0.236								0.381
Dry sub-season, not averaged	ABGR	-0.895	0.171	-1.012	0.215	-1.448	-2.416	1.273	0.280				0.208	-0.235	-0.149	0.129	0.328		-3.628						0.276
	LAOC	-1.469	0.265	-0.912	0.079				0.249	-2.900			0.325	-0.748			0.487								0.290
	PIMO	-1.187	0.387	-0.876	0.158	-0.834	-1.661		0.445				0.433		-0.127		0.270		-3.192	-7.312					0.464
	PIPO	-1.058	0.338	-0.845	0.129		-1.553		0.350				0.369		-0.146		0.221								0.368
	PSME	-1.266	0.131	-1.261	0.153				0.180			-2.741	0.251				0.348								0.273
	THPL	-0.648	0.151	-1.109	0.299	-1.170			0.289				0.274				0.312								0.370
Dry sub-season, averaged within days	ABGR	-1.094	0.643	-0.933	0.394	-0.779			0.603		-2.008		0.218	-4.011	-2.433	2.232	0.462	-6.184	-2.708						0.748
	LAOC	-1.277	0.625	-1.049	0.283				0.598				0.250				0.711								0.724
	PIMO	-1.104	0.449	-0.954	0.201				0.514				0.167				0.149	-9.173	-5.642	-16.055		17.085	10.089	-11.346	0.569
	PIPO	-1.100	0.698	-0.945	0.226				0.684				0.157		-3.357		0.259								0.670
	PSME	-0.930	0.455	-0.950	0.490				0.594			-2.933	0.282		-1.797		0.476								0.646
	THPL	-0.642	0.267	-0.987	0.349	-1.336			0.361				0.312				0.388								0.313

Supplemental Table 1. Coefficients of simple and multiple linear regressions shown are significant at $p<0.05$. The datasets used are listed on the left. Data "averaged within days" consists of means of 3-4 trees' parameters within species each hour. "Not averaged" data occurs only in dry sub-season and relates each individual tree's vapor pressure deficit (VPD), leaf water potential (Ψ_{leaf}), and/or predawn water potential (Ψ_{PD}). VPDs were in kPa; Ψ_{leaf} and Ψ_{PD} values were in bars; and g_s were in mol m^{-2} s^{-1}.

Supplemental Text 1:

Samples that included mature shoots were consistently around 0.6 MPa more negative than samples comprised entirely of new growth, a pattern that was not seen in the other species current vs. previous year growth. We speculate that there may be greater resistance to water going to the more mature *L. occidentalis* foliage, effectively shunting water to the developing leaves and xylem in the distal portion of the branch and necessitating more negative water potentials in the mature sections to maintain transpiration. The relatively little new growth in June of 2015, compared to the following months, could account for why similarly sized samples would contain more mature shoots. If more mature shoots depressed Ψ_{leaf}, the later water potential measurements that did not include mature shoots in the sample overestimated the water potentials in *L. occidentalis.* Another potential explanation could be the immaturity of the needles on the *L. occidentalis* samples. For the other species, second year needles were sampled when current year needles were not yet hardened. For the deciduous *L. occidentalis*, however, first year samples were used because they were the only needles present.
